# Insights into the New Molecular Updates in Acute Myeloid Leukemia Pathogenesis

**DOI:** 10.3390/genes14071424

**Published:** 2023-07-10

**Authors:** Derya Demir

**Affiliations:** Department of Pathology, Ege University Faculty of Medicine, Izmir 35100, Turkey; derya.demir@ege.edu.tr

**Keywords:** acute myeloid leukemia, pathogenesis, molecular updates, genomics

## Abstract

As our understanding of the biologic basis of acute myeloid leukemia evolves, so do the classification systems used to describe this group of cancers. Early classification systems focused on the morphologic features of blasts and other cell populations; however, the explosion in genomic technologies has led to rapid growth in our understanding of these diseases and thus the refinement of classification systems. Recently, two new systems, the International Consensus Classification system and the 5th edition of the World Health Organization classification of tumors of hematopoietic and lymphoid tissues, were published to incorporate the latest genomic advances in blood cancer. This article reviews the major updates in acute myeloid leukemia in both systems and highlights the biologic insights that have driven these changes.

## 1. Introduction

Acute myeloid leukemia (AML) is a heterogeneous group of hematopoietic malignancies characterized by a proliferation of immature cells (blasts). Early classification systems were based primarily on the morphologic features of these blasts, while in the last two decades, the predominant classification systems by the World Health Organization (WHO) have increasingly incorporated immunophenotypic and genetic characteristics to refine these groupings. The last major update to this classification occurred in 2016 with the revised 4th edition of the WHO classification of tumors of hematopoietic and lymphoid tissues [1].

The revised 4th edition WHO classification system (WHO4R) included several genetically defined subtypes of AML, specifically “AML with recurrent genetic abnormalities (AML-RGA)”, a subset of “AML with myelodysplasia-related changes (AML-MRC)”, and “AML with germline predisposition” [1]. Since the publication of this system, genomic testing has rapidly expanded, and with it, our understanding of the genetic underpinnings of AML. This more nuanced understanding has catalyzed efforts to refine the classification systems, which have been undertaken both by the WHO as part of the WHO 5th edition classification [2] and by the Society of Hematopathology (SH), European Association for Hematopathology (EAHP), and Clinical Advisory Committees (CAC) as the international consensus classification (ICC) [3,4].

These two classifications, the 5th edition of the WHO (WHO5) and the ICC, retain much of the overall structure of the prior AML genetic subtypes, with the persistence of the above three categories based on (1) recurrent genetic abnormalities, (2) myelodysplasia-associated genetic changes, albeit both new classifications recognize specific mutations as myelodysplasia-related, and (3) germline predisposition. While these classifications maintain the same structure as the prior, they include additions, refinements, and removals of entities present in the prior system. This article will review the major updates in the genetically defined groups of AML and discuss the underlying biology of these specifically updated entities. The specific changes to blast thresholds described in each system are outside the scope of this article but are briefly discussed for cases with the *RUNX1* mutation. Throughout this article, the ICC classification will be listed before the WHO5 classification based on alphabetical order alone and is not meant to suggest preferential endorsement of either system.

## 2. AML with Recurrent Genetic Abnormalities

The WHO4R system identified a group of AMLs defined by specific gene abnormalities as “AML with recurrent genetic abnormalities (AML-RGA)”. ICC retains this name for the group, while WHO5 renames the group “*AML with defining genetic abnormalities (AML-DGA)*”. Within AML-DGA, the WHO5 also includes a placeholder category, “AML with other defined alterations”. The specific entities within these systems are elaborated in Table 1.

### 2.1. RUNX1 Mutations

The *RUNX1* gene encodes a core binding factor transcription factor that is essential to hematopoietic differentiation and is frequently altered in hematopoietic neoplasms by both germline and somatic alterations, including point mutations and translocation [5]. Both new classification systems have removed the WHO4R entity “AML with mutated *RUNX1*” as the authors felt this alteration did not define a significantly distinct disease entity given the potential for misclassification of “AML with germline predisposition (*RUNX1* mutation)” and the close association with progression from myelodysplastic syndromes (MDS) and prior chemoradiotherapy [3,6,7,8]. The ICC has recognized *RUNX1* mutations as myelodysplasia-related in AML, and therefore, cases of myeloid neoplasms with ≥10–19% blasts in the bone marrow are defined as MDS/AML, while cases with ≥20% blasts are defined as AML with myelodysplasia-related gene mutations (*RUNX1*). The WHO5 would classify cases with ≥20% blasts as AML and provide subtyping based on differentiation in the absence of other specific genetic alterations.

### 2.2. RARA, KMT2A, and MECOM Rearrangements

Several oncogenic drivers were recognized as translocation partners by the WHO4R, with the most common partner as the defining RGA and variant partners listed as variants of the primary entity. ICC continues to recognize the specific entities of “acute promyelocytic leukemia (APL) with t(15;17) (q24.1;q21.2)/*PML::RARA*”, “AML with t(9;11) (p21.3;q23.3)/*MLLT3::KMT2A*”, and “AML with inv(3) (q21.3q26.2) or t(3;3) (q21.3;q26.2)/*GATA2; MECOM(EVI1)*” as distinct entities; and also recognizes other leukemias with similar biology but driven by variant fusion partners as “APL with other *RARA* rearrangements”, “AML with other *KMT2A* rearrangements”, and “AML with other *MECOM* rearrangements” [3,4]. WHO5 does not alter the classification of APL from WHO4R; however, it replaces the specific entities of “AML with t(9;11) (p21.3;q23.3)/*MLLT3::KMT2A*” and “AML with inv(3) (q21.3q26.2) or t(3;3) (q21.3;q26.2)/*GATA2; MECOM(EVI1)*” with the more generic driver-translocated groups denoted in the ICC [2]. The expansion of these diagnostic groups is anticipated to allow more standardized reporting of AML with these driver alterations and non-canonical fusion partners.

### 2.3. CEBPA Mutations

**Figure 1:**
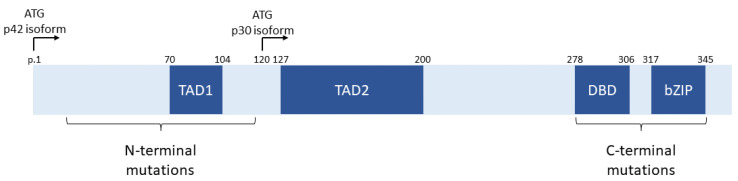
Schematic diagram depicting the *CEBPA* gene structure and distribution of mutations. ATG p42 and p30 denote the locations of the initiation codon for the p42 and p30 isoforms of CEBPA, respectively. TAD1—transactivation domain 1; TAD2—transactivation domain 2; DBD—DNA binding domain; bZIP—basic leucine zipper domain. p. positions refer to the protein position in the p42 isoform encoded by NCBI transcript NM_004364.

The CCAAT/enhancer binding protein α (C/EBP-α) transcription factor is encoded by the *CEBPA* gene on the long arm of chromosome 19. This transcription factor is an essential regulator of granulopoiesis, and multiple isoforms are encoded by the *CEBPA* gene [9]. The long isoform, p42, is encoded by the first ATG initiation codon, while the shorter p30 isoform is encoded by a downstream ATG, as shown in Figure 1 [9,10,11]. Both isoforms contain a DNA binding domain (DBD) and a basic leucine zip (bZIP) dimerization domain at the C-terminus of the protein. The p30 isoform contains only the second transactivation domain (TAD2), while the p42 isoform contains both transactivation domains (TAD1 and TAD2). Mutations in *CEBPA* tend to cluster in two regions: In the N-terminus of the protein upstream of the p30 initiation codon and in the C-terminus in the DBD and bZIP domains [10,11]. The N-terminal mutations tend to be nonsense and frameshift mutations and prevent translation of intact p42 isoforms but do not affect p30 [11,12]. The C-terminal mutations (now termed bZIP mutations despite occurring both within the bZIP and DBD domains) are generally missense or in-frame insertions or deletions leading to altered function [11,12].

Numerous studies of *CEBPA* mutations in AML found an improved prognosis in patients with biallelic mutations, with each allele harboring a mutation from each of these clusters [13,14,15]. This finding led to the WHO4R entity of “AML with biallelic mutation of *CEBPA*.” [1]. In addition, germline *CEBPA* mutations have been identified in families with inherited risk of AML (WHO4R “AML with germline predisposition (*CEBPA* mutation)”) [1,16], and these mutations tend to be N-terminal nonsense and frameshift alterations. A subset of AML with biallelic mutations on further analysis was found to be acquired C-terminal mutations in patients with a germline N-terminal alteration [12,17]. More recently, two large studies in adults and children have shown that the positive prognostic impact of *CEBPA* mutations is most correlated with C-terminal (aka bZIP) mutations, either as part of a biallelic alteration or alone [18,19]. Because of the findings of these large studies, the de novo subtypes of *CEBPA*-mutated AML in the new classification systems have been updated to “AML with in-frame bZIP *CEBPA* mutations” in ICC and “AML with *CEBPA* mutation” in WHO5, with the caveat that in WHO5, the acceptable mutations are either biallelic (*biCEBPA*) or single mutations in the bZIP domain (*smbZIP-CEBPA*). A large proportion of clinical sequencing in AML is performed using next-generation sequencing, where it is technically challenging to assess the phase of such distant mutations as the N- and C- terminal alterations in *CEBPA*. Thus, the updated classification, by removing a requirement for biallelic alterations, will clarify which patients belong in this favorable prognostic group.

### 2.4. TP53 Alterations

Located on the short arm of chromosome 17, the *TP53* gene encodes the tumor suppressor P53 and is the most commonly mutated gene in cancer [20,21]. P53 has been referred to as the “guardian of the genome” due to its key role in several pathways, including DNA damage response, cell cycle arrest, senescence, and apoptosis [22]. *TP53* alterations have been well documented to occur in myeloid neoplasms, including MDS and AML, where they act through a dominant-negative or loss-of-function mechanism [23]. Patients with myeloid malignancies harboring *TP53* alterations have a very poor prognosis, especially those with multiple simultaneous *TP53* alterations [24,25,26]. The uniquely poor clinical outcomes in *TP53*-altered patients have led to numerous updates in the new classification systems.

The ICC now recognizes an entity of “AML with mutated *TP53*” as a distinct subtype, whereas WHO5 has not yet included this as a unique group, despite noting the association of *TP53* alterations with very poor outcomes (Table 1) [2,3,4]. Although the systems diverge in the case of *TP53* mutation, they both recognize copy number changes affecting *TP53* as myelodysplasia-related cytogenetic abnormalities (i.e., monosomy 17, deletion of 17p, isochromosome 17q, and other abnormalities leading to deletion or loss of 17p, see Table 2) [2,3,4]. Similarly, both systems now also recognize germline *TP53* mutations as germline predisposition events for the development of leukemia (Table 3) [2,3,4]. By including this new entity of particularly aggressive disease, it is expected that all new patients will be assessed for *TP53* mutation status upfront to properly predict their clinical course.

### 2.5. Other Rare Recurrent Genetic Alterations

Both the ICC and WHO5 identify additional rare subtypes of AML with recurrent alterations; however, there are too many entities included within these lists to fully elaborate in the current manuscript [2,3,4]. Among this group, *NUP98* rearrangements are highlighted as similar to rearrangements involving *RARA*, *KMT2A*, and *MECOM*; ICC recognizes distinct fusion partners and a generic group of “AML with *NUP98* and other partners”, while WHO5 only recognizes the generic group of “AML with *NUP98* rearrangement.” [2,3,4]. While this is a heterogeneous group within and between the classification systems, its inclusion and subtypes will encourage the recognition of specific genetic subgroups and enable standardized reporting and data collection to better understand the biology of these entities.

## 3. Myelodysplasia-Related AML

The concept of myelodysplasia-related AML has been retained from the WHO4R grouping of “AML with myelodysplasia-related changes (AML-MRC)”, although there are now specific additional genetic alterations recognized as “myelodysplasia-related defining”, and both systems have removed the criteria of morphologic dysplasia for classification in this group (Table 2) [1,2,3,4]. These defining genomic criteria included updated cytogenetic changes as well as newly defined mutations, which were wholly absent in the WHO4R.

As noted above in the section on *TP53* alterations, 17p abnormalities have been added as defining criteria for myelodysplasia-related AML in both ICC and WHO5. Also common to both systems is the removal of several specific translocations from the summary classification systems released to date [2,3,4]. ICC has notably added trisomy 8 and deletion of 20q to this group of cytogenetic changes, while WHO5 adds deletion of 11q [2,3,4].

The most significant change to this category of myelodysplasia-related AML is the new inclusion of specific somatic mutations in many of the most recurrently mutated genes in MDS [27,28,29,30], but it excludes the common clonal-hematopoiesis-related mutations *DNMT3A* and *TET2* [31,32,33]. The list of somatically mutated genes is largely shared between the systems and includes *ASXL1*, *BCOR*, *EZH2*, *SF3B1*, *SRSF2*, *STAG2*, *U2AF1*, and *ZRSR2.* As noted above in the section on the *RUNX1* mutation, this is also considered myelodysplasia-related in the ICC but not in WHO5 [2,3,4]. These genes are critical components of several cell processes, including transcription (*RUNX1*), epigenetics/gene expression (*ASXL1*, *BCOR*, and *EZH2*), DNA replication/integrity (*STAG2*), and RNA splicing (*SF3B1*, *SRSF2*, *U2AF1*, and *ZRSR2*).

Overall, the changes within this category of AML now emphasize the genetic correlates of the poor prognosis in myelodysplasia-related AML while removing the reliance on morphology to define this group.

## 4. AML with Germline Predisposition

The last broad group of genetically defined AML is associated with germline predisposition. This group was initially included in the WHO4R and has been retained in both the ICC and the WHO5, although the new systems recognize a few additional entities and expand previously defined categories (Table 3) [1,2,3,4,34]. For brevity, this section will only review the newly included entities and omit the specifically enumerated bone marrow failure syndromes, updated terminology and subtyping of juvenile myelomonocytic leukemia (JMML)-associated syndromes, and germline *TP53* alterations, the latter of which were briefly mentioned in the AML-RGA section above. By recognizing several new entities, it is hoped that patients at higher risk for disease will be more closely followed and related donors appropriately screened in cases of allogeneic stem cell transplant.

### 4.1. Germline SAMD9/SAMD9L Mutation

**Figure 2:**
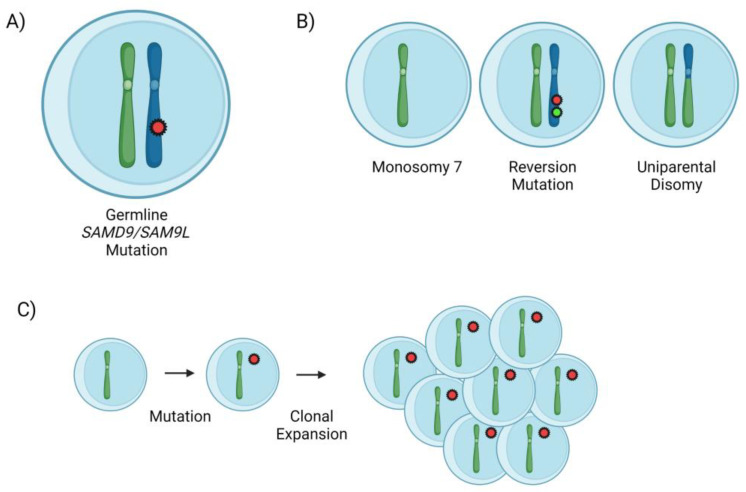
Pathogenic mechanism of progression in germline *SAMD9/SAMD9L* mutation. (**A**) Hematopoietic stem cell harboring germline gain-of-function *SAMD9/SAMD9L* mutation on chromosome 7. (**B**) In response to cellular stress, there is selective pressure for reversion via loss of chromosome 7 (monosomy), reversion mutation of *SAMD9/SAMD9L*, or uniparental disomy in the region of the mutation. (**C**) Cells that lose chromosome 7 are at risk of acquiring additional hits and progressing to a myeloid neoplasm. Created with BioRender.com.

*SAMD9* and *SAMD9L* are two adjacent genes located on the long arm of chromosome 7 at 7q21 [35]. The exact function of their encoded proteins, SAMD9 and SAMD9L, is unclear; however, they are both believed to have an anti-proliferative function in normal cells [36]. Germline gain-of-function mutations in each gene are associated with a specific abnormal clinical phenotype; *SAMD9* mutations are associated with the MIRAGE (myelodysplasia, infection, restriction of growth, adrenal hypoplasia, genital phenotypes, enteropathy) syndrome, while *SAMD9L* mutations are associated with an ataxia pancytopenia (ATXPC) syndrome [35,36,37,38]. It is hypothesized that in the setting of stress (i.e., infection or inflammation), there is selective pressure to overcome the hypo-proliferative effect of the germline *SAMD9/SAMD9L* mutation, which occurs predominantly through loss of the affected chromosome 7, although it may also occur through a secondary reversion mutation in the mutated gene or uniparental disomy of the wild-type allele in the region of the mutation [35,39,40]. Subsequently, cells that acquire monosomy 7 will not carry the hyperactive *SAMD9/SAMD9L* allele but may acquire additional somatic mutations leading to progression to MDS and AML (see Figure 2) [35,39,40]. In addition, the loss of the mutant allele following the loss of chromosome 7 will lead to a false-negative result if only neoplastic cells are tested for *SAMD9/SAMD9L* mutations.

### 4.2. Biallelic Germline BLM Mutation/Bloom Syndrome

Bloom syndrome is a rare autosomal recessive disorder characterized by short stature, photosensitivity with rash, and an increased risk of solid and hematologic cancers [41,42]. This syndrome is caused by mutations in the *BLM* gene on the long arm of chromosome 15, which encodes the BLM protein, a RecQ DNA helicase protein [41,42]. The BLM helicase is a critical component of normal DNA replication, and loss of function leads to genomic instability with a high incidence of chromosome breaks [41,43]. This syndrome and its associated increased risk of AML is a new distinct entity in WHO5 and is discussed under the group of “Additional conditions with germline predisposition to hematologic malignancy and provisional entities” in ICC [2,3,34].

## 5. Discussion

In recent years, molecular and genetic alterations have played an essential role in improving the prognosis of AML. Induction therapies (typically anthracycline and cytarabine-based) have eliminated leukemic blasts in AML. However, as our understanding of the molecular pathogenesis of the disease has improved, new therapeutic approaches have become crucial, especially in relapsed/refractory AML [44]. At this juncture, personalized therapeutic approaches assume prominence, emphasizing the need for tailored interventions based on individual patient characteristics. Incorporating cytogenetic changes alongside other prognostic factors for cases classified in the unfavorable risk group becomes crucial in determining the optimal treatment strategy. Notably, allogeneic hematopoietic stem cell transplantation (Allo-HSCT) is often considered a therapeutic option in these cases, specifically during the initial attainment of complete remission [45,46]. Patients who experience relapse are generally classified within the unfavorable prognostic group, regardless of their cytogenetic profile. However, most AMLs exhibit a normal karyotype, placing them in the intermediate cytogenetic risk group. Within this group, some demonstrate inadequate responses to standard chemotherapy consolidation treatments. Consequently, there is a pressing need to classify the intermediate-risk group based on novel biomarkers, enabling more precise risk stratification and selecting appropriate therapeutic interventions [47,48]. Targeted therapies take precedence in the group of patients harboring mutations in *FLT3*, *NPM1*, *KIT*, *CEBPA*, and *TET2*, which contribute to leukemogenesis. Risk groups in AML are classified into three categories according to the 2022 European Leukemia Net (ELN) risk stratification based on genetics [49].

*1. Favorable risk: prognostic entities in **bold** are newly defined in ELN 2022.*
*-t(8;21)(q22;q22.1)/RUNX1::RUNX1T1*
*-inv(16)(p13.1;q22) or t(16;16)(p13.1;q22)/CBFB::MYH1*
  The risk category remains unchanged, regardless of *KIT* or *FLT3* mutations.
  -***Mutated NPM1 without FLT3-ITD***
  AML with *NPM1* mutations and adverse risk cytogenetic abnormalities is classified as adverse risk [50]. The specific role of additional molecular abnormalities, except for *FLT3-ITD*, in patients with *NPM1*-mutated AML remains undefined.
  -***bZIP in-frame mutated CEBPA***
  In-frame mutations within the *bZIP* region of *CEBPA* have explicitly been associated with a favorable outcome, regardless of whether they occur as monoallelic or biallelic mutations.
*2. Intermediate risk: prognostic entities in **bold** are newly defined in ELN 2022.*
  -***FLT-ITD (regardless of allelic ratio or NPM1 mutation)***
  The importance of the allelic ratio of *FLT3-ITD* has diminished in this group due to the lack of standardized measurement techniques [51,52]. Regardless of the allelic ratio and *NPM1* mutation status, this group falls into the intermediate-risk category. Midostaurin therapy has significant importance in this group.
  -*t(9;11)(p21.3;q23.3)/MLLT3::KMT2A*
  The presence of *t(9;11)(p21.3;q23.3)* takes priority over rare concurrent adverse-risk gene mutations.
  -Cytogenetic and/or molecular abnormalities not classified as favorable or adverse
*3. Adverse risk: prognostic entities in **bold** are newly defined in ELN 2022.*
*-t(6;9)(p23;q34.1)/DEK::NUP214*
*-t(v;11q23.3)/KMT2A rearranged (excluding KMT2A-PTD)*
*-t(9;22)(q34.1;q11.2)/BCR::ABL1*
***-(8;16)(p11;p13)/KAT6A::CREBBP***
  Furthermore, these adverse-risk cytogenetic abnormalities, such as *t(3q26.2;v)* involving the *MECOM* gene and *t(8;16)(p11;p13)* associated with *KAT6A::CREBBP*, have demonstrated a dismal long-term overall survival. Allo-HSCT is a potentially beneficial treatment option in such cases [53,54].
*-inv(3)(q21.3;q26.2) or t(3;3)(q21.3;q26.2)/GATA2, MECOM (EVI1)*
***-t(3q26.2;v)/MECOM(EVI1)-rearranged***
  -Monosomy 5 or del(5q); monosomy 7; monosomy 17/abn(17p)
-***Complex karyotype (Complex karyotype, defined as the presence of three or more unrelated chromosome abnormalities without other class-defining recurring genetic abnormalities, excludes hyperdiploid karyotypes with three or more trisomies (or polysomies) in the absence of structural abnormalities), monosomal karyotype***
  Moreover, hyperdiploid karyotypes characterized by multiple trisomies (or polysomies) are now excluded from the complex karyotype classification and the adverse risk group. This reclassification is based on the observation that patients with numerical cytogenetic changes and the absence of structural abnormalities have shown better survival outcomes than those with three or more cytogenetic changes accompanied by structural abnormalities [55].
  -***Mutated*** *ASXL1, **BCOR, EZH2,** RUNX1, **SF3B1, SRSF2, STAG2, U2AF1, or ZRSF2***
  -*Mutated TP53* ***(Variant Allele Frequency ≥ 10%)***

### Treatments and Future Directions

In newly diagnosed AML, intensive chemotherapy is administered if no targetable mutations are present. However, in *FLT3*-mutated AML, midostaurin (quizartinib investigational) is added to intensive chemotherapy. In therapy-related AML and myelodysplasia-related AML, CPX351 (hypomethylating agents and venetoclax investigational) is preferred. CPX351, a liposomal formulation of daunorubicin and cytarabine, received approval in 2017 for treating therapy-related AML and myelodysplasia-related AML. For cases with complex karyotypes and *TP53* mutations, ongoing research is investigating hypomethylating agents and venetoclax as alternative treatment options [49]. Gemtuzumab ozogamicin (GO) is a monoclonal antibody that selectively targets CD33, a cell surface antigen expressed on leukemic blasts in AML. It is conjugated to the cytotoxic agent calicheamicin and released upon binding to CD33, leading to the elimination of CD33-expressing leukemia cells. Studies have shown that adding GO to standard AML therapy can benefit patients with favorable and possibly intermediate-risk newly diagnosed AML [49]. Venetoclax is a BH3 mimetic drug that inhibits the pro-apoptotic protein BCL2, leading to apoptosis in AML [56]. In recent developments, venetoclax has received approval for use in combination with hypomethylating agents (HMAs) or low-dose cytarabine (LDAC) for patients unsuitable for intensive chemotherapy for newly diagnosed AML patients. The hedgehog pathway, an important signaling pathway during embryonic development, is overexpressed in myeloid blasts [57]. This observation has led to the evaluation of the hedgehog pathway inhibitor glasdegib in treating AML [57].

The clinical investigation of new therapies and novel combinations plays a vital role in further enhancing the outcomes of AML [58]. Exploring drug development strategies beyond single-agent dose-finding studies is essential, particularly in the relapsed setting. This approach has resulted in the successful approval of targeted therapies such as *FLT3*, *IDH1*, and *IDH2* inhibitors. Currently, menin inhibitors are being evaluated as a treatment option for patients with *KMT2A* rearrangements or *NPM1* mutations, following the same assessment pattern for their efficacy and safety [59,60,61,62]. *MLL* (mixed-lineage leukemia) translocations involving the *KMT2A* gene at chromosome 11q23 are present in approximately 5% to 10% of adults with AML [63]. *MLL* translocations result in the dysregulation and increased expression of homeobox (*HOX*) genes. These genes are also dysregulated in AML cases with *NPM1* mutations and are associated with the self-renewal capacity of hematopoietic stem cells [64]. The scaffold protein menin, encoded by the *MEN1* gene, plays a crucial role in the function of *KMT2A* [65]. Menin binds to *KMT2A* and is essential for its activity. In recent years, small-molecule inhibitors that interfere with the interaction between *KMT2A* and menin have been under investigation. Two such inhibitors are SNDX 5613 (AUGMENT 101 trial) and KO539 (KOMET1 trial). These inhibitors have shown efficacy in early-phase trials, with SNDX 5613 demonstrating composite complete response rates of 44% among 45 patients with *NPM1* or *MLL*-rearranged AML [59,66].

While there have been significant advancements in the management of AML, the outcomes for patients with high-risk diseases remain unsatisfactory. Numerous clinical trials are currently underway to improve these outcomes, evaluating targeted therapies and immunotherapies as potential treatment options. These trials investigate using these therapies as monotherapies or in combination with other treatment modalities. Although most of these therapies are still in the investigational stage and long-term outcomes are not yet fully described, some promising early responses have been observed. In this context, uproleselan (GM-1271) is a drug that functions as an inhibitor of *E-selectin*. It has been discovered that uproleselan can disrupt vascular niche-mediated chemoresistance. In the context of cancer, the vascular niche refers to the microenvironment surrounding blood vessels, which can provide a protective environment for cancer cells and contribute to their resistance to chemotherapy [67,68].

CD47 is a signaling molecule known as a “*do not eat me*” signal, which is overexpressed by cancer cells [69]. It serves as an antiphagocytic signal, allowing cancer cells to evade phagocytosis by macrophages, a process by which immune cells engulf and eliminate harmful cells. This immune evasion mechanism enables cancer cells to escape the immune system. Magrolimab is a monoclonal antibody that explicitly targets CD47. By binding to CD47 on cancer cells, magrolimab blocks the signal, effectively removing the protection against phagocytosis [70]. This enhances the ability of macrophages to recognize and engulf cancer cells, leading to their elimination.

MBG453, or sabatolimab, is a humanized antibody designed to target a protein called *TIM3*. *TIM3* acts as an inhibitory checkpoint on immune cells and blasts, regulating immune responses. Significantly, *TIM3* is not expressed on normal hematopoietic stem cells. Further research and clinical trials are necessary to understand the efficacy of MBG453 and determine its role in treating AML and high-risk MDS. Nonetheless, these preliminary findings suggest that targeting *TIM3* with sabatolimab holds promise as a therapeutic strategy for patients with AML [71,72].

In addition to sabatolimab and magrolimab, several other immune therapies are currently under development for treating AML. These therapies include bispecific antibodies and chimeric antigen receptor (CAR) T-cell therapy, targeting various antigens such as CD123, CD33, and CD70 [73,74]. Flotetuzumab is one of the most clinically advanced immunotherapeutic approaches. It is a bispecific antibody-based molecule known as a *DART* (dual affinity re-targeting) antibody. Flotetuzumab is designed to bind to CD3ε on T cells simultaneously and CD123 on AML blasts. Doing so redirects the immune system to recognize and eliminate AML cells [75].

Developing bispecific antibodies and CAR T-cell therapies represents a promising field in AML treatment. These therapies aim to enhance the body’s immune response against leukemia cells, potentially improving outcomes for patients with AML. Ongoing research and clinical trials will further evaluate the safety and efficacy of these immunotherapeutic approaches [76]. One challenge in developing immune therapies for AML is the lack of a leukemia-specific or dispensable antigen on AML blasts, such as CD19 in acute lymphoblastic leukemia. While CD33 and CD123 are abundantly expressed on AML blasts, they are also present on standard hematopoietic stem and progenitor cells, which can lead to significant myelotoxicity when targeted by therapies [75].

Researchers are exploring innovative approaches such as genetic editing using *CRISPR/Cas9* technology to address this issue. One strategy involves editing out the CAR target antigen from a donor allograft, creating a donor graft with a modified antigen profile that is now “leukemia specific”. After Allo-HSCT, CAR T-cells can be administered, explicitly targeting the modified antigen [76]. This approach allows CAR T-cell persistence and anti-leukemic activity while minimizing prolonged myeloablation and potential harm to normal hematopoietic cells. However, the success of such an approach in clinical trials is still under investigation, and further research is needed to determine its feasibility and effectiveness.

Overall, developing effective and safe immunotherapeutic strategies for AML remains a complex and evolving area of research, and ongoing clinical trials will provide valuable insights into the potential of these innovative approaches.

## 6. Conclusions

Since the last update to the classification of AML in 2016 with the WHO4R, there has been a huge growth in our understanding of the pathobiology and, in particular, the genomics of this group of diseases. The updated classification systems, while harboring minor differences, both incorporate these changes to refine specific AML entities. Through efforts to more clearly differentiate patients with different disease biology, we gain the ability to better tailor therapies and deliver personalized care in acute myeloid leukemia.

## Figures and Tables

**Table 1:** Acute myeloid leukemia (AML) with recurrent/defining genetic abnormalities.

WHO4R	ICC	WHO5
**AML with recurrent genetic abnormalities**	**AML with recurrent genetic abnormalities**	**AML with defining genetic abnormalities**
AML with t(8;21)(q22;q22.1); *RUNX1-RUNX1T1*	AML with t(8;21) (q22;q22.1)/*RUNX1::RUNX1T1*	AML with *RUNX1::RUNX1T1* fusion
AML with inv(16)(p13.1q22) or t(16;16)(p13.1;q22); *CBFB-MYH11*	AML with inv(16)(p13.1q22) or t(16;16) (p13.1;q22)/*CBFB::MYH11*	AML with *CBFB::MYH11* fusion
APL with *PML-RARA*	APL with t(15;17) (q24.1;q21.2)/*PML::RARA*	APL with *PML::RARA* fusion
	**APL with other *RARA* rearrangements**	
AML with t(9;11)(p21.3;q23.3); *KMT2A-MLLT3*	AML with t(9;11) (p21.3;q23.3)/*MLLT3::KMT2A*	**AML with *KMT2A* rearrangement**
	**AML with other *KMT2A* rearrangements**	
AML with t(6;9)(p23;q34.1); *DEK-NUP214*	AML with t(6;9) (p22.3;q34.1)/*DEK::NUP214*	AML with *DEK::NUP214* fusion
AML with inv(3)(q21.3q26.2) or t(3;3)(q21.3;q26.2); *GATA2*, *MECOM*	AML with inv(3) (q21.3q26.2) or t(3;3) (q21.3;q26.2)/*GATA2; MECOM(EVI1)*	**AML with *MECOM* rearrangement**
	**AML with other *MECOM* rearrangements**	
AML (megakaryoblastic) with t(1;22) (p13.3;q13.1); *RBM15-MKL1*	AML (megakaryoblastic) with t(1;22) (p13.3;q13.1)/*RBM15::MRTF1*	AML with *RBM15::MRTFA* fusion
AML with *BCR-ABL1*	AML with t(9;22) (q34.1;q11.2)/*BCR::ABL1*	AML with *BCR::ABL1* fusion
AML with biallelic mutation of *CEBPA*	**AML with in-frame bZIP *CEBPA* mutations**	**AML with *CEBPA* mutation**
AML with mutated *NPM1*	AML with mutated *NPM1*	AML with *NPM1* mutation
AML with mutated *RUNX1* *	**AML with myelodysplasia-related gene mutations (*RUNX1*)**	**AML, defined by differentiation**
	**AML with mutated *TP53***	
	**AML with t(5;11) (q35.2;p15.4/ *NUP98::NSD1***	**AML with *NUP98* rearrangement**
	**AML with t(11;12) (p15.4;p13.3)/*NUP98::KMD5A***	
	**AML with *NUP98* and other partners**	
	**AML with inv(16) (p13.3q24.3)/*CBFA2T3::GLIS2***	**AML with other defined genetic alterations (*CBFA2T3::GLIS2*)**
	**AML with t(1;3) (p36.3;q21.3)/*PRDM16::RPN1***	
	**AML with t(3;5) (q25.3;q35.1)/*NPM1::MLF1***	
	**AML with t(8;16) (p11.2;p13.3)/*KAT6A::CREBBP***	
	**AML with t(7;12) (q36.3;p13.2)/*ETV6::MNX1***	
	**AML with t(10;11) (p12.3;q14.2)/*PICALM::MLLT10***	
	**AML with t(16;21) (p11.2;q22.2)/*FUS::ERG***	
	**AML with t(16;21) (q24.3;q22.1)/*RUNX1::CBFA2T3***	

AML—acute myeloid leukemia; APL—acute promyelocytic leukemia; WHO4R—revised 4th edition of the World Health Organization classification; ICC—international consensus classification; WHO5—5th edition of the World Health Organization classification; diagnostic entities in **bold** are newly defined in the ICC and/or WHO5 classification systems; * denotes diagnoses removed from the WHO4R.

**Table 2:** Myelodysplasia-related acute myeloid leukemia (AML).

WHO4R	ICC	WHO5
AML with myelodysplasia-related changes	**AML with myelodysplasia-related gene mutations (AML-MRGM)**	**AML, myelodysplasia-related** Defining somatic mutations
	***ASXL1***	***ASXL1***
	***BCOR***	***BCOR***
	***EZH2***	***EZH2***
	***RUNX1***	
	***SF3B1***	***SF3B1***
	***SRSF2***	***SRSF2***
	***STAG2***	***STAG2***
	***U2AF1***	***U2AF1***
	***ZRSR2***	***ZRSR2***
Defining cytogenetic abnormalities	**AML with myelodysplasia-related cytogenetic abnormalities (AML-MRCA)**	**AML, myelodysplasia-related ** Defining cytogenetic abnormalities
Complex karyotype (≥ 3 abnormalities)	Complex karyotype (≥3 unrelated clonal chromosomal abnormalities)	Complex karyotype (≥3 abnormalities)
del(5q) or t(5q)	del(5q)/t(5q)/add(5q)	5q deletion or loss of 5q due to unbalanced translocation
Loss of chromosome 7 or del(7q)	−7/del(7q)	Monosomy 7, 7q deletion, or loss of 7q due to unbalanced translocation
	**+8**	
del(11q)		11q deletion
del(12p) or t(12p)	del(12p)/t(12p)/add(12p)	12p deletion or loss of 12p due to unbalanced translocation
Loss of chromosome 13 or del(13q)		Monosomy 13 or 13q deletion
	**−17/add(17p) or del(17p)**	**17p deletion or loss of 17p due to unbalanced translocation**
Isochromosome 17q or t(17p)	i(17q)	Isochromosome 17q
idic(X)(q13)	idic(X)(q13)	idic(X)(q13)
	**del(20q)**	
t(11;16)(q23.3;p13.3) *		
t(3;21)(q26.2;q22.1) *		
t(1;3)(p36.3;q21.2) *		
t(2;11)(p21;q23.3) *		
t(5;12)(q32;p13.2) *		
t(5;7)(q32;q11.2) *		
t(5;17)(q32;p13.2) *		
t(5;10)(q32;q21) *		
t(3;5)(q25.3;q35.1) *		

AML—acute myeloid leukemia; WHO4R—revised 4th edition of the World Health Organization classification; ICC—international consensus classification; WHO5—5th edition of the World Health Organization classification; diagnostic entities in **bold** are newly defined in the ICC and/or WHO5 classification systems; * denotes diagnoses removed from the WHO4R.

**Table 3:** Acute myeloid leukemia (AML) associated with germline predisposition.

WHO4R	ICC	WHO5
Myeloid neoplasms with germline predisposition without a pre-existing disorder or organ dysfunction	Hematologic neoplasms with germline predisposition without a constitutional disorder affecting multiple organ systems	Myeloid neoplasms with germline predisposition without a preexisting platelet disorder or organ dysfunction
AML with germline CEBPA mutation	Myeloid neoplasms with germline CEBPA mutation	Germline CEBPA P/LP variant (CEBPA-associated familial AML)
Myeloid neoplasms with germline DDX41 mutation	Myeloid or lymphoid neoplasms with germline DDX41 mutation	Germline DDX41 P/LP variant
	Myeloid or lymphoid neoplasms with germline TP53 mutation	Germline TP53 P/LP variant (Li-Fraumeni syndrome)
		
Myeloid neoplasms with germline predisposition and pre-existing platelet disorders	Hematologic neoplasms with germline predisposition associated with a constitutional platelet disorder	Myeloid neoplasms with germline predisposition and pre-existing platelet disorder
Myeloid neoplasms with germline RUNX1 mutation	Myeloid or lymphoid neoplasms with germline RUNX1 mutation	Germline RUNX1 P/LP variant (familial platelet disorder with associated myeloid malignancy, FPD-MM)
Myeloid neoplasms with germline ANKRD26 mutation	Myeloid neoplasms with germline ANKRD26 mutation	Germline ANKRD26 P/LP variant (Thrombocytopenia 2)
Myeloid neoplasms with germline ETV6 mutation	Myeloid or lymphoid neoplasms with germline ETV6 mutation	Germline ETV6 P/LP variant (Thrombocytopenia 5)
		
Myeloid neoplasms with germline predisposition and other organ dysfunction	Hematologic neoplasms with germline predisposition associated with a constitutional disorder affecting multiple organ systems	Myeloid neoplasms with germline predisposition and potential organ dysfunction
Myeloid neoplasms with germline GATA2 mutation	Myeloid neoplasms with germline GATA2 mutation	Germline GATA2 P/LP variant (GATA2-deficiency)
	Myeloid neoplasms with germline SAMD9 mutation	Germline SAMD9 P/LP variant (MIRAGE Syndrome)
	Myeloid neoplasms with germline SAMD9L mutation	Germline SAMD9L P/LP variant (SAMD9L-related Ataxia Pancytopenia Syndrome)
Myeloid neoplasms associated with bone marrow failure syndromes	Myeloid neoplasms associated with bone marrow failure syndromes	Bone marrow failure syndromes
	○ Fanconi anemia	○ Fanconi anemia (FA)
	○ Shwachman-Diamond syndrome	○ Shwachman-Diamond syndrome (SDS)
	○ Severe congenital neutropenia	○ Severe congenital neutropenia (SCN)
	○ Diamond-Blackfan anemia	
Myeloid neoplasms associated with telomere biology disorders	Telomere biology disorders including dyskeratosis congenita	Telomere biology disorders
Juvenile myelomonocytic leukemia associated with neurofibromatosis, Noonan syndrome, or Noonan syndrome-like disorders	JMML associated with neurofibromatosis	RASopathies (Neurofibromatosis type 1, CBL syndrome, Noonan syndrome, or Noonan syndrome-like disorders)
	JMML associated with Noonan-syndrome-like disorder (CBL-syndrome)	
Myeloid neoplasms associated with Down syndrome	Myeloid or lymphoid neoplasms associated with Down syndrome	Down syndrome
		Biallelic germline BLM P/LP variant (Bloom syndrome)

AML—acute myeloid leukemia; WHO4R—revised 4th edition of the World Health Organization classification; ICC—international consensus classification; WHO5—5th edition of the World Health Organization classification; P/LP—pathogenic/likely pathogenic; diagnostic entities in **bold** are newly defined in the ICC and/or WHO5 classification systems.

## Data Availability

Not applicable.
